# 6-Ethyl-*N*-methyl-3-nitro-4-nitro­methyl-4*H*-chromen-2-amine

**DOI:** 10.1107/S1600536811053554

**Published:** 2011-12-21

**Authors:** J. Muthukumaran, A. Parthiban, H. Surya Prakash Rao, R. Krishna

**Affiliations:** a Centre for Bioinformatics, Pondicherry University, Puducherry 605 014, India; b Department of Chemistry, Pondicherry University, Puducherry 605 014, India

## Abstract

In the title compound, C_13_H_15_N_3_O_5_, the O and N atoms of the nitro­methyl group and the methyl C atom of the ethyl group are disordered over two sets of sites with refined occupancies of 0.629 (7):0.371 (7) and 0.533 (8):0.467 (8), respectively. The dihydro­pyran ring has an extremely flattened conformation. An intra­molecular N—H⋯O hydrogen bond occurs. In the crystal, pairs of N—H⋯O hydrogen bonds link mol­ecules, forming inversion dimers. In addition, weak inter­molecular C—H⋯O hydrogen bonds are also present.

## Related literature

For the biological and pharmacological importance of 4*H-*chromene derivatives, see: Cai (2007 ▶, 2008 ▶); Cai *et al.* (2006 ▶); Gabor (1988 ▶); Brooks (1998 ▶); Hyana & Saimoto (1987 ▶); Tang *et al.* (2007 ▶). For related structures, see: Muthukumaran *et al.* (2011*a* ▶,*b* ▶,*c* ▶); Gayathri *et al.* (2006 ▶); Bhaskaran *et al.* (2006 ▶).
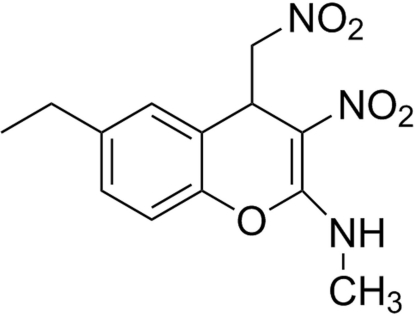


## Experimental

### 

#### Crystal data

C_13_H_15_N_3_O_5_
*M* *_r_* = 293.28
Triclinic, 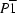

*a* = 8.2538 (10) Å
*b* = 9.0431 (9) Å
*c* = 10.3323 (12) Å
α = 73.484 (9)°
β = 71.728 (11)°
γ = 83.234 (9)°
*V* = 701.75 (14) Å^3^
*Z* = 2
Mo *K*α radiation
μ = 0.11 mm^−1^
*T* = 293 K
0.4 × 0.35 × 0.2 mm

#### Data collection

Oxford Diffraction Xcalibur Eos diffractometer
Absorption correction: multi-scan (*CrysAlis PRO*; Oxford Diffraction, 2009 ▶) *T*_min_ = 0.958, *T*_max_ = 0.979
4281 measured reflections
2463 independent reflections
1520 reflections with *I* > 2σ(*I*)
*R*_int_ = 0.031

#### Refinement

*R*[*F* ^2^ > 2σ(*F* ^2^)] = 0.073
*wR*(*F* ^2^) = 0.232
*S* = 1.06
2463 reflections
205 parameters
122 restraints
H-atom parameters constrained
Δρ_max_ = 0.40 e Å^−3^
Δρ_min_ = −0.34 e Å^−3^

### 

Data collection: *CrysAlis PRO* (Oxford Diffraction, 2009 ▶); cell refinement: *CrysAlis PRO*; data reduction: *CrysAlis PRO*; program(s) used to solve structure: *SHELXS97* (Sheldrick, 2008 ▶); program(s) used to refine structure: *SHELXL97* (Sheldrick, 2008 ▶); molecular graphics: *ORTEP-3 for Windows* (Farrugia, 1997 ▶); software used to prepare material for publication: *PLATON* (Spek, 2009 ▶).

## Supplementary Material

Crystal structure: contains datablock(s) I, global. DOI: 10.1107/S1600536811053554/lh5388sup1.cif

Structure factors: contains datablock(s) I. DOI: 10.1107/S1600536811053554/lh5388Isup2.hkl

Supplementary material file. DOI: 10.1107/S1600536811053554/lh5388Isup3.cml

Additional supplementary materials:  crystallographic information; 3D view; checkCIF report

## Figures and Tables

**Table 1:** Hydrogen-bond geometry (Å, °)

*D*—H⋯*A*	*D*—H	H⋯*A*	*D*⋯*A*	*D*—H⋯*A*
N1—H1⋯O2	0.86	1.97	2.600 (3)	129
N1—H1⋯O2^i^	0.86	2.21	2.943 (4)	143
C11—H11*A*⋯O3^ii^	0.97	2.58	3.258 (4)	128
C12—H12*A*⋯O2^iii^	0.97	2.55	3.457 (5)	156

Symmetry codes: (i) 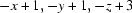; (ii) 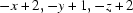; (iii) 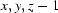.

**Fig. 1.**
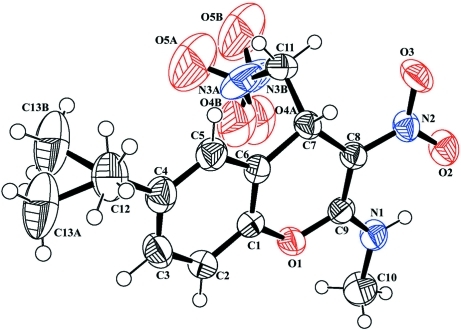
: The molecular structure of (I), showing displacement ellipsoids drawn at the 50% probability level.

**Fig. 2.**
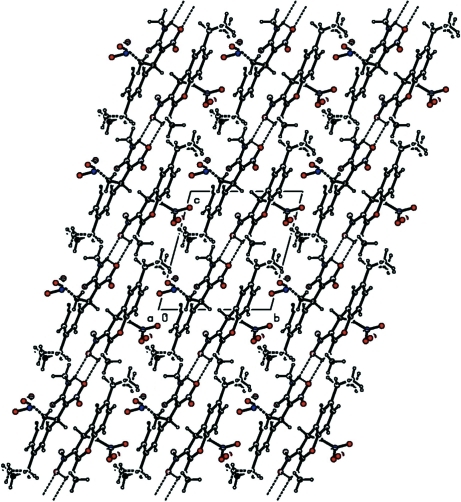
: The crystal packing of (I) showing intermolecular hydrogen bonds as dashed lines.

## supplementary crystallographic information

### Comment

The 4*H-*chromene moiety frequently appears as a main structural component in various biologically important compounds. They exhibit the various pharmacological properties such as anti-coagulant, anti-viral, anti-fungal, anti-inflammatory, anti-diabetic and anti-cancer activity (Cai, 2008; Cai, 2007; Cai *et al.*, 2006; Gabor *et al.*, 1988; Brooks, 1998; Hyana & Saimoto, 1987; Tang *et al.*, 2007). Considering the growing medicinal importance of these derivatives, an X-ray crystallographic study on the title compound was carried out. In the molecular structure of the title compound (I) (Fig. 1) the O and N atoms of the nitromethyl group and the methyl C atom of the ethyl group are disordered over two sets of sites with refined occupancies of 0.629 (7):0.371 (7) and 0.533 (8):0.467 (8), respectively. Some crystal structures of related 4*H-*chromene derivatives have already been published e.g. *N*-methyl-3-nitro-4-(nitromethyl)-4*H*-chromen-2-amine (Muthukumaran *et al.*, 2011*c*), 6,8-dichloro-*N*-methyl-3-nitro-4-nitro-methyl-4*H*-chromen-2-amine (Muthukumaran *et al.*, 2011*a*), 6-methoxy-*N*-methyl-3-nitro-4-nitromethyl-4*H*-chromen-2-amine (Muthukumaran *et al.*, 2011*b*), *N*-benzyl-*N*-[4-methylsulfanyl)-3-nitro-4*H*-chromen-2-yl] amine (Bhaskaran *et al.*, 2006) and *N*,6-dimethyl-4-(methylsulfanyl)-3-nitro-4*H*-chromen-2-amine (Gayathri *et al.*, 2006). In the crystal, N—H···O hydrogen bonds form centrosymmetric dimers (Fig .2). In addition, there are weak intermolecular C—H···O hydrogen bonds.

### Experimental

To a solution of (*E*)-5-ethyl-2-(2-nitrovinyl)phenol (150 mg, 0.77 mmol) in methanol (5 mL), 1,8-diazabicyclo[5.4.0]undec-7-ene (15 mg, 0.10 mmol) was added and stirred for 10 minutes at room temperature. To this solution (*E*) *N*-methyl-1-(methylthio)-2-nitroethenamine (115 mg, 0.77 mmol) was added and stirred for 8 h until completion of the reaction (TLC, hexane:ethyl acetate, 3:2, *R*f = 1/2). The reaction mixture was then kept aside at 278 K in a refrigerator for 3 h to afford racemic mixture of the product as a white precipitate, which was filtered. Good crystals were obtained by recrystallization of a solution of dichloromethane: hexane (9:3 *v*/*v*).

### Refinement

H atoms were positioned geometrically (C—H = 0.93–0.98 Å. N—H = 0.86Å) and were refined using a riding model with *U*_iso_(H) = xU_eq_(C,N), where *x* = 1.5 for methyl and 1.2 for all other atoms. The nitro and terminal carbon atom of ethyl group are disordered over two orientations, with the refined site-occupancy ratios being 0.629 (7):0.371 (7) and 0.533 (8):0.467 (8), respectively. The *DFIX*, SIMU, DELU and EADP commands in *SHELXL* (Sheldrick, 2008) were used to model the disorder.

### Crystal data

**Table:**

C_13_H_15_N_3_O_5_	*Z* = 2
*M**_r_* = 293.28	*F*(000) = 308
Triclinic, *P*1	*D*_x_ = 1.388 Mg m^−^^3^
Hall symbol: -P 1	Mo *K*α radiation, λ = 0.71073 Å
*a* = 8.2538 (10) Å	Cell parameters from 1935 reflections
*b* = 9.0431 (9) Å	θ = 2.7–29.1°
*c* = 10.3323 (12) Å	µ = 0.11 mm^−^^1^
α = 73.484 (9)°	*T* = 293 K
β = 71.728 (11)°	Block, colorless
γ = 83.234 (9)°	0.4 × 0.35 × 0.2 mm
*V* = 701.75 (14) Å^3^	

### Data collection

**Table:**

Oxford Diffraction Xcalibur Eos diffractometer	2463 independent reflections
Radiation source: fine-focus sealed tube	1520 reflections with *I* > 2σ(*I*)
graphite	*R*_int_ = 0.031
Detector resolution: 15.9821 pixels mm^-1^	θ_max_ = 25.0°, θ_min_ = 2.7°
ω scans	*h* = −9→9
Absorption correction: multi-scan (*CrysAlis PRO*; Oxford Diffraction, 2009)	*k* = −10→10
*T*_min_ = 0.958, *T*_max_ = 0.979	*l* = −11→12
4281 measured reflections	

### Refinement

**Table:**

Refinement on *F*^2^	Primary atom site location: structure-invariant direct methods
Least-squares matrix: full	Secondary atom site location: difference Fourier map
*R*[*F*^2^ > 2σ(*F*^2^)] = 0.073	Hydrogen site location: inferred from neighbouring sites
*wR*(*F*^2^) = 0.232	H-atom parameters constrained
*S* = 1.06	*w* = 1/[σ^2^(*F*_o_^2^) + (0.1377*P*)^2^ + 0.1022*P*] where *P* = (*F*_o_^2^ + 2*F*_c_^2^)/3
2463 reflections	(Δ/σ)_max_ < 0.001
205 parameters	Δρ_max_ = 0.40 e Å^−^^3^
122 restraints	Δρ_min_ = −0.34 e Å^−^^3^

### Special details

**Table:**

Geometry. All e.s.d.'s (except the e.s.d. in the dihedral angle between two l.s. planes) are estimated using the full covariance matrix. The cell e.s.d.'s are taken into account individually in the estimation of e.s.d.'s in distances, angles and torsion angles; correlations between e.s.d.'s in cell parameters are only used when they are defined by crystal symmetry. An approximate (isotropic) treatment of cell e.s.d.'s is used for estimating e.s.d.'s involving l.s. planes.
Refinement. Refinement of *F*^2^ against ALL reflections. The weighted *R*-factor *wR* and goodness of fit *S* are based on *F*^2^, conventional *R*-factors *R* are based on *F*, with *F* set to zero for negative *F*^2^. The threshold expression of *F*^2^ > σ(*F*^2^) is used only for calculating *R*-factors(gt) *etc*. and is not relevant to the choice of reflections for refinement. *R*-factors based on *F*^2^ are statistically about twice as large as those based on *F*, and *R*- factors based on ALL data will be even larger.

### Fractional atomic coordinates and isotropic or equivalent isotropic displacement parameters (Å^2^)

**Table:**

	*x*	*y*	*z*	*U*_iso_*/*U*_eq_	Occ. (<1)
O1	0.4139 (2)	0.2857 (3)	1.1942 (2)	0.0468 (6)	
C1	0.5007 (4)	0.2700 (4)	1.0581 (3)	0.0401 (8)	
C8	0.6569 (4)	0.4021 (4)	1.1982 (3)	0.0383 (8)	
O2	0.6415 (3)	0.5176 (3)	1.3724 (2)	0.0569 (7)	
O3	0.8792 (3)	0.5209 (3)	1.2039 (2)	0.0636 (8)	
N2	0.7266 (3)	0.4819 (3)	1.2608 (3)	0.0435 (7)	
C9	0.4856 (4)	0.3561 (4)	1.2598 (3)	0.0394 (8)	
N1	0.3831 (3)	0.3738 (3)	1.3790 (3)	0.0490 (8)	
H1	0.4224	0.4167	1.4267	0.059*	
C6	0.6684 (4)	0.3088 (4)	0.9922 (3)	0.0380 (8)	
C2	0.4066 (4)	0.2138 (4)	0.9941 (4)	0.0490 (9)	
H2	0.2935	0.1877	1.0408	0.059*	
C5	0.7416 (4)	0.2899 (4)	0.8563 (3)	0.0489 (9)	
H5	0.8551	0.3151	0.8099	0.059*	
C7	0.7700 (4)	0.3607 (4)	1.0681 (3)	0.0413 (8)	
H7	0.8238	0.4563	1.0044	0.050*	
C4	0.6519 (5)	0.2354 (4)	0.7888 (4)	0.0528 (9)	
C3	0.4833 (5)	0.1970 (4)	0.8600 (4)	0.0548 (10)	
H3	0.4209	0.1592	0.8160	0.066*	
C11	0.9145 (4)	0.2466 (4)	1.0924 (4)	0.0588 (10)	
H11A	0.9964	0.2440	1.0021	0.071*	
H11B	0.9726	0.2804	1.1468	0.071*	
N3A	0.8506 (10)	0.0891 (5)	1.1687 (9)	0.085 (2)	0.629 (7)
O4A	0.7196 (14)	0.0755 (17)	1.2654 (17)	0.113 (3)	0.629 (7)
O5A	0.9064 (12)	−0.0297 (9)	1.1366 (11)	0.169 (3)	0.629 (7)
N3B	0.8580 (16)	0.0962 (10)	1.1944 (16)	0.085 (2)	0.371 (7)
O4B	0.717 (2)	0.043 (3)	1.240 (3)	0.113 (3)	0.371 (7)
O5B	0.9759 (19)	0.0179 (16)	1.2268 (18)	0.169 (3)	0.371 (7)
C12	0.7376 (6)	0.2240 (6)	0.6386 (4)	0.0778 (13)	
H12A	0.7279	0.3246	0.5751	0.093*	0.533 (8)
H12B	0.8581	0.2008	0.6277	0.093*	0.533 (8)
H12C	0.6550	0.2506	0.5857	0.093*	0.467 (8)
H12D	0.8286	0.2970	0.5932	0.093*	0.467 (8)
C13A	0.6702 (15)	0.1062 (12)	0.5913 (10)	0.114 (3)	0.533 (8)
H13A	0.7334	0.1097	0.4951	0.171*	0.533 (8)
H13B	0.5517	0.1291	0.5983	0.171*	0.533 (8)
H13C	0.6828	0.0050	0.6506	0.171*	0.533 (8)
C13B	0.8100 (18)	0.0627 (8)	0.6369 (12)	0.114 (3)	0.467 (8)
H13D	0.8636	0.0583	0.5411	0.171*	0.467 (8)
H13E	0.7197	−0.0094	0.6804	0.171*	0.467 (8)
H13F	0.8929	0.0369	0.6881	0.171*	0.467 (8)
C10	0.2057 (4)	0.3261 (6)	1.4383 (4)	0.0722 (13)	
H10A	0.1380	0.3912	1.3825	0.108*	
H10B	0.1624	0.3350	1.5336	0.108*	
H10C	0.2001	0.2209	1.4376	0.108*	

### Atomic displacement parameters (Å^2^)

**Table:**

	*U*^11^	*U*^22^	*U*^33^	*U*^12^	*U*^13^	*U*^23^
O1	0.0348 (11)	0.0592 (17)	0.0485 (13)	−0.0089 (10)	−0.0032 (9)	−0.0247 (11)
C1	0.0371 (16)	0.041 (2)	0.0445 (17)	0.0043 (14)	−0.0130 (14)	−0.0158 (14)
C8	0.0367 (16)	0.039 (2)	0.0394 (16)	−0.0034 (13)	−0.0094 (13)	−0.0109 (14)
O2	0.0646 (15)	0.0694 (19)	0.0409 (12)	−0.0110 (13)	−0.0074 (11)	−0.0262 (12)
O3	0.0457 (14)	0.085 (2)	0.0667 (16)	−0.0271 (13)	−0.0008 (12)	−0.0383 (15)
N2	0.0472 (16)	0.0450 (18)	0.0393 (14)	−0.0071 (12)	−0.0081 (12)	−0.0155 (13)
C9	0.0362 (16)	0.0365 (19)	0.0431 (17)	0.0004 (13)	−0.0080 (14)	−0.0114 (14)
N1	0.0431 (15)	0.058 (2)	0.0448 (15)	−0.0076 (13)	0.0022 (12)	−0.0255 (14)
C6	0.0404 (17)	0.0360 (19)	0.0386 (16)	0.0007 (13)	−0.0114 (13)	−0.0126 (14)
C2	0.0420 (18)	0.050 (2)	0.063 (2)	0.0021 (15)	−0.0210 (16)	−0.0223 (18)
C5	0.0472 (18)	0.051 (2)	0.0468 (19)	−0.0022 (16)	−0.0073 (15)	−0.0175 (16)
C7	0.0361 (16)	0.047 (2)	0.0416 (17)	−0.0084 (14)	−0.0050 (13)	−0.0173 (15)
C4	0.064 (2)	0.052 (2)	0.0476 (19)	0.0046 (17)	−0.0212 (17)	−0.0185 (17)
C3	0.066 (2)	0.050 (2)	0.063 (2)	0.0010 (18)	−0.0350 (19)	−0.0208 (18)
C11	0.0356 (17)	0.080 (3)	0.074 (2)	0.0052 (17)	−0.0169 (16)	−0.042 (2)
N3A	0.075 (3)	0.079 (3)	0.095 (4)	0.044 (2)	−0.033 (2)	−0.023 (2)
O4A	0.131 (3)	0.045 (7)	0.154 (7)	−0.008 (3)	−0.030 (3)	−0.021 (4)
O5A	0.189 (6)	0.098 (5)	0.222 (9)	0.055 (5)	−0.065 (5)	−0.065 (5)
N3B	0.075 (3)	0.079 (3)	0.095 (4)	0.044 (2)	−0.033 (2)	−0.023 (2)
O4B	0.131 (3)	0.045 (7)	0.154 (7)	−0.008 (3)	−0.030 (3)	−0.021 (4)
O5B	0.189 (6)	0.098 (5)	0.222 (9)	0.055 (5)	−0.065 (5)	−0.065 (5)
C12	0.100 (3)	0.084 (3)	0.056 (2)	−0.003 (3)	−0.022 (2)	−0.031 (2)
C13A	0.168 (10)	0.096 (6)	0.073 (5)	−0.015 (6)	0.000 (5)	−0.049 (4)
C13B	0.168 (10)	0.096 (6)	0.073 (5)	−0.015 (6)	0.000 (5)	−0.049 (4)
C10	0.046 (2)	0.092 (4)	0.073 (3)	−0.015 (2)	0.0122 (19)	−0.041 (2)

### Geometric parameters (Å, °)

**Table:**

O1—C9	1.342 (4)	C11—N3B	1.4847 (11)
O1—C1	1.403 (3)	C11—N3A	1.4847 (11)
C1—C6	1.375 (4)	C11—H11A	0.9700
C1—C2	1.380 (4)	C11—H11B	0.9700
C8—N2	1.366 (4)	N3A—O4A	1.2113 (11)
C8—C9	1.414 (4)	N3A—O5A	1.2115 (11)
C8—C7	1.496 (4)	N3B—O4B	1.2114 (11)
O2—N2	1.256 (3)	N3B—O5B	1.2117 (11)
O3—N2	1.256 (3)	C12—C13B	1.5122 (11)
C9—N1	1.298 (4)	C12—C13A	1.5125 (11)
N1—C10	1.463 (4)	C12—H12A	0.9700
N1—H1	0.8600	C12—H12B	0.9700
C6—C5	1.395 (4)	C12—H12C	0.9700
C6—C7	1.501 (4)	C12—H12D	0.9700
C2—C3	1.376 (5)	C13A—H13A	0.9600
C2—H2	0.9300	C13A—H13B	0.9600
C5—C4	1.372 (5)	C13A—H13C	0.9600
C5—H5	0.9300	C13B—H13D	0.9600
C7—C11	1.517 (5)	C13B—H13E	0.9600
C7—H7	0.9800	C13B—H13F	0.9600
C4—C3	1.387 (5)	C10—H10A	0.9600
C4—C12	1.518 (5)	C10—H10B	0.9600
C3—H3	0.9300	C10—H10C	0.9600
			
C9—O1—C1	120.9 (2)	O4A—N3A—O5A	114.3 (8)
C6—C1—C2	122.1 (3)	O4A—N3A—C11	118.5 (9)
C6—C1—O1	122.2 (3)	O5A—N3A—C11	126.8 (8)
C2—C1—O1	115.7 (3)	O4B—N3B—O5B	118.9 (19)
N2—C8—C9	120.1 (3)	O4B—N3B—C11	128.9 (19)
N2—C8—C7	117.6 (2)	O5B—N3B—C11	112.1 (11)
C9—C8—C7	122.2 (3)	C13B—C12—C13A	53.2 (7)
O3—N2—O2	119.8 (3)	C13B—C12—C4	110.9 (5)
O3—N2—C8	118.7 (2)	C13A—C12—C4	116.9 (5)
O2—N2—C8	121.5 (3)	C13B—C12—H12A	141.0
N1—C9—O1	113.2 (3)	C13A—C12—H12A	108.1
N1—C9—C8	126.7 (3)	C4—C12—H12A	108.1
O1—C9—C8	120.1 (3)	C13B—C12—H12B	59.8
C9—N1—C10	125.0 (3)	C13A—C12—H12B	108.1
C9—N1—H1	117.5	C4—C12—H12B	108.1
C10—N1—H1	117.5	H12A—C12—H12B	107.3
C1—C6—C5	117.5 (3)	C13B—C12—H12C	109.5
C1—C6—C7	120.6 (3)	C13A—C12—H12C	57.4
C5—C6—C7	121.8 (3)	C4—C12—H12C	109.5
C3—C2—C1	118.7 (3)	H12A—C12—H12C	56.1
C3—C2—H2	120.6	H12B—C12—H12C	142.2
C1—C2—H2	120.6	C13B—C12—H12D	109.5
C4—C5—C6	122.2 (3)	C13A—C12—H12D	133.6
C4—C5—H5	118.9	C4—C12—H12D	109.5
C6—C5—H5	118.9	H12A—C12—H12D	55.3
C8—C7—C6	111.4 (2)	H12B—C12—H12D	53.9
C8—C7—C11	114.3 (3)	H12C—C12—H12D	108.0
C6—C7—C11	111.6 (3)	C12—C13A—H13A	109.5
C8—C7—H7	106.3	C12—C13A—H13B	109.5
C6—C7—H7	106.3	H13A—C13A—H13B	109.5
C11—C7—H7	106.3	C12—C13A—H13C	109.5
C5—C4—C3	118.2 (3)	H13A—C13A—H13C	109.5
C5—C4—C12	119.3 (3)	H13B—C13A—H13C	109.5
C3—C4—C12	122.5 (3)	C12—C13B—H13D	109.5
C2—C3—C4	121.4 (3)	C12—C13B—H13E	109.5
C2—C3—H3	119.3	H13D—C13B—H13E	109.5
C4—C3—H3	119.3	C12—C13B—H13F	109.5
N3B—C11—C7	114.4 (6)	H13D—C13B—H13F	109.5
N3A—C11—C7	111.4 (4)	H13E—C13B—H13F	109.5
N3B—C11—H11A	117.3	N1—C10—H10A	109.5
N3A—C11—H11A	109.3	N1—C10—H10B	109.5
C7—C11—H11A	109.3	H10A—C10—H10B	109.5
N3B—C11—H11B	97.5	N1—C10—H10C	109.5
N3A—C11—H11B	109.3	H10A—C10—H10C	109.5
C7—C11—H11B	109.3	H10B—C10—H10C	109.5
H11A—C11—H11B	108.0		
			
C9—O1—C1—C6	7.7 (5)	C1—C6—C7—C8	−14.8 (4)
C9—O1—C1—C2	−172.0 (3)	C5—C6—C7—C8	168.7 (3)
C9—C8—N2—O3	179.8 (3)	C1—C6—C7—C11	114.3 (3)
C7—C8—N2—O3	2.4 (4)	C5—C6—C7—C11	−62.2 (4)
C9—C8—N2—O2	−0.7 (5)	C6—C5—C4—C3	−0.6 (5)
C7—C8—N2—O2	−178.2 (3)	C6—C5—C4—C12	177.5 (3)
C1—O1—C9—N1	174.0 (3)	C1—C2—C3—C4	0.0 (5)
C1—O1—C9—C8	−6.3 (4)	C5—C4—C3—C2	0.4 (6)
N2—C8—C9—N1	−4.2 (5)	C12—C4—C3—C2	−177.5 (4)
C7—C8—C9—N1	173.1 (3)	C8—C7—C11—N3B	59.4 (9)
N2—C8—C9—O1	176.1 (3)	C6—C7—C11—N3B	−68.2 (9)
C7—C8—C9—O1	−6.6 (5)	C8—C7—C11—N3A	72.3 (5)
O1—C9—N1—C10	−1.1 (5)	C6—C7—C11—N3A	−55.3 (5)
C8—C9—N1—C10	179.2 (4)	N3B—C11—N3A—O4A	65 (4)
C2—C1—C6—C5	0.3 (5)	C7—C11—N3A—O4A	−41.2 (12)
O1—C1—C6—C5	−179.4 (3)	N3B—C11—N3A—O5A	−123 (4)
C2—C1—C6—C7	−176.3 (3)	C7—C11—N3A—O5A	131.0 (10)
O1—C1—C6—C7	4.0 (5)	N3A—C11—N3B—O4B	−64 (4)
C6—C1—C2—C3	−0.4 (5)	C7—C11—N3B—O4B	15 (2)
O1—C1—C2—C3	179.3 (3)	N3A—C11—N3B—O5B	112 (4)
C1—C6—C5—C4	0.2 (5)	C7—C11—N3B—O5B	−169.6 (13)
C7—C6—C5—C4	176.8 (3)	C5—C4—C12—C13B	96.2 (7)
N2—C8—C7—C6	−166.3 (3)	C3—C4—C12—C13B	−85.9 (8)
C9—C8—C7—C6	16.4 (4)	C5—C4—C12—C13A	154.5 (7)
N2—C8—C7—C11	66.0 (4)	C3—C4—C12—C13A	−27.5 (8)
C9—C8—C7—C11	−111.3 (3)		

### Hydrogen-bond geometry (Å, °)

**Table:**

*D*—H···*A*	*D*—H	H···*A*	*D*···*A*	*D*—H···*A*
N1—H1···O2	0.86	1.97	2.600 (3)	129
N1—H1···O2^i^	0.86	2.21	2.943 (4)	143
C11—H11A···O3^ii^	0.97	2.58	3.258 (4)	128
C12—H12A···O2^iii^	0.97	2.55	3.457 (5)	156

Symmetry codes:  (i) −*x*+1, −*y*+1, −*z*+3; (ii) −*x*+2, −*y*+1, −*z*+2; (iii) *x*, *y*, *z*−1.
